# Supplementary material for: Effects of Astaxanthin from Shrimp Shell on Oxidative Stress and Behavior in Animal Model of Alzheimer’s Disease
Source: Mar Drugs. 2019 Nov 4;17(11):628. doi: 10.3390/md17110628 (PMC6891431; doi:10.3390/md17110628)
Supplement: Supplementary file 1 [file marinedrugs-17-00628-s001.pdf]

Supplementary

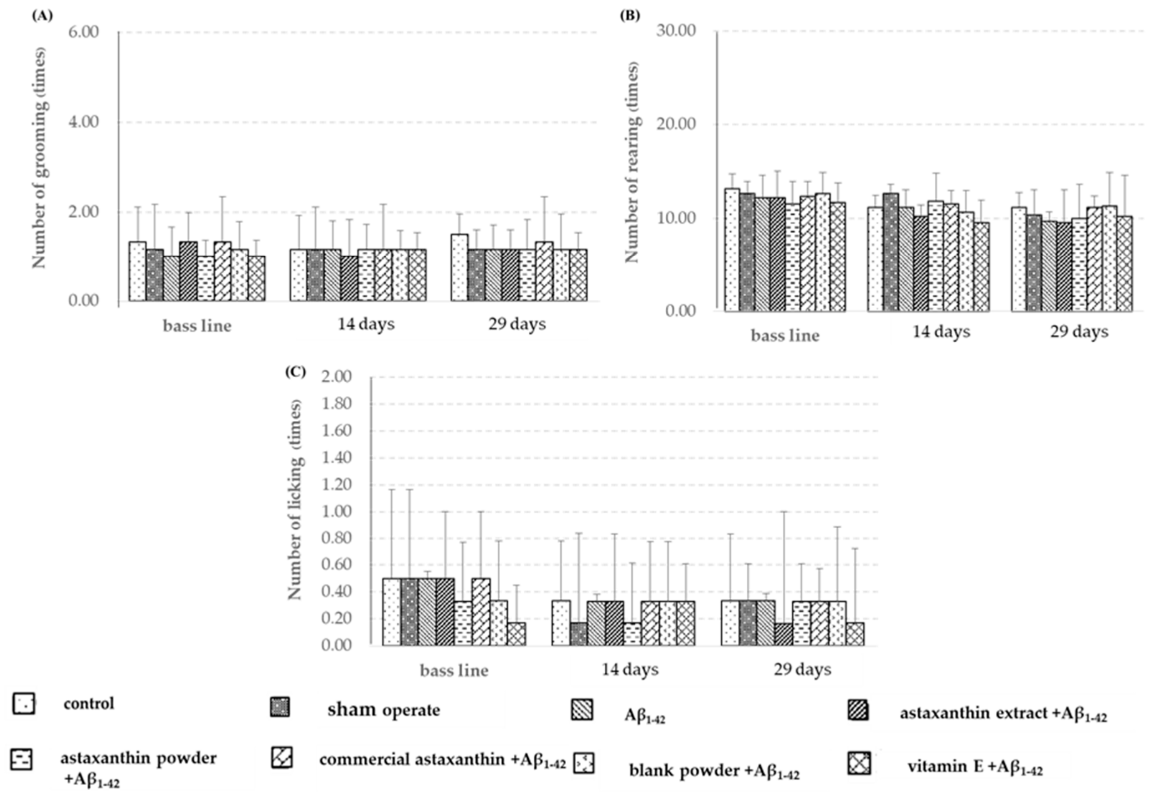

Figure S1. Effect of astaxanthin on spontaneous motor behaviors including grooming (**A**), rearing (**B**) and licking behaviors (**C**). in control rats (**C**), sham operate (SO): ACSF or vehicle plus $A\beta_{1-42}$ treated group (V), vitamin E: 100 mg/kg BW plus $A\beta_{1-42}$ treated group (VE), astaxanthin extract: 10 mg/kg BW plus $A\beta_{1-42}$ treated group (AE), commercial astaxanthin: 10 mg/kg BW plus $A\beta_{1-42}$ treated group (AC), astaxanthin powder: 10 mg/kg BW plus $A\beta_{1-42}$ treated group (AP) and blank powder: 10 mg/kg BW plus $A\beta_{1-42}$ treated group (BP).

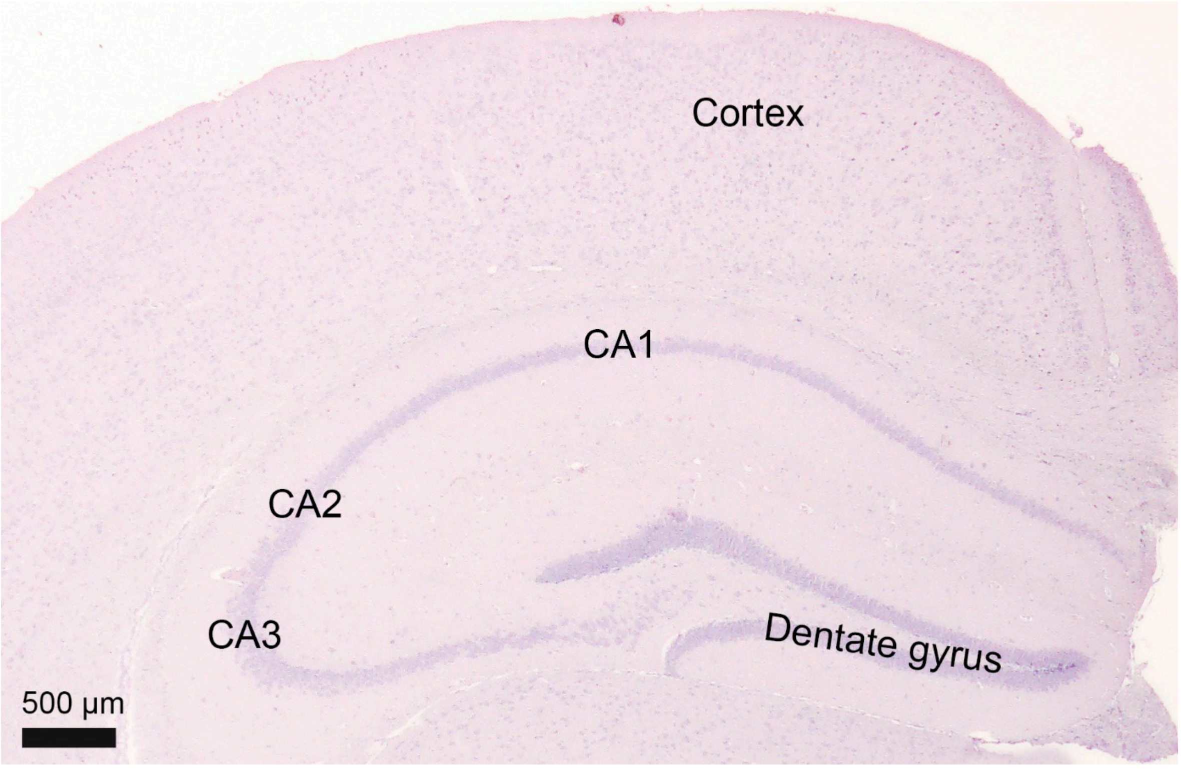

Figure S2. Hematoxylin staining of the paraffin-embedded right mouse brain demonstrates CA1, CA2, CA3, dentate gyrus, and cortex regions.

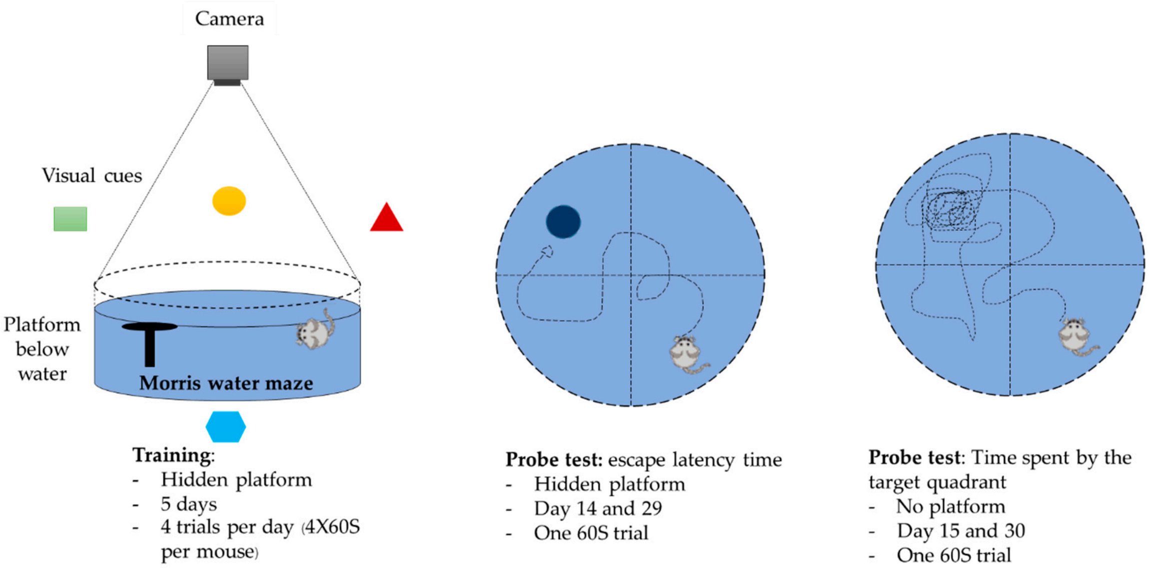

Figure S3. Schematic of Morris water maze test

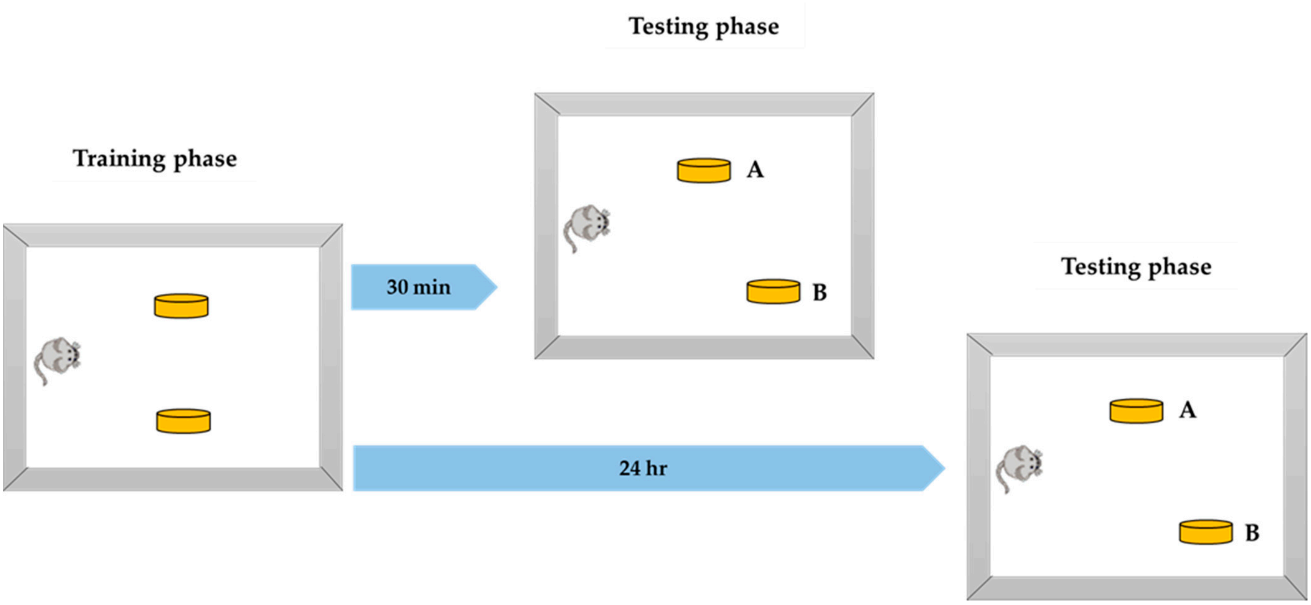

Figure S4. Schematic of object location test

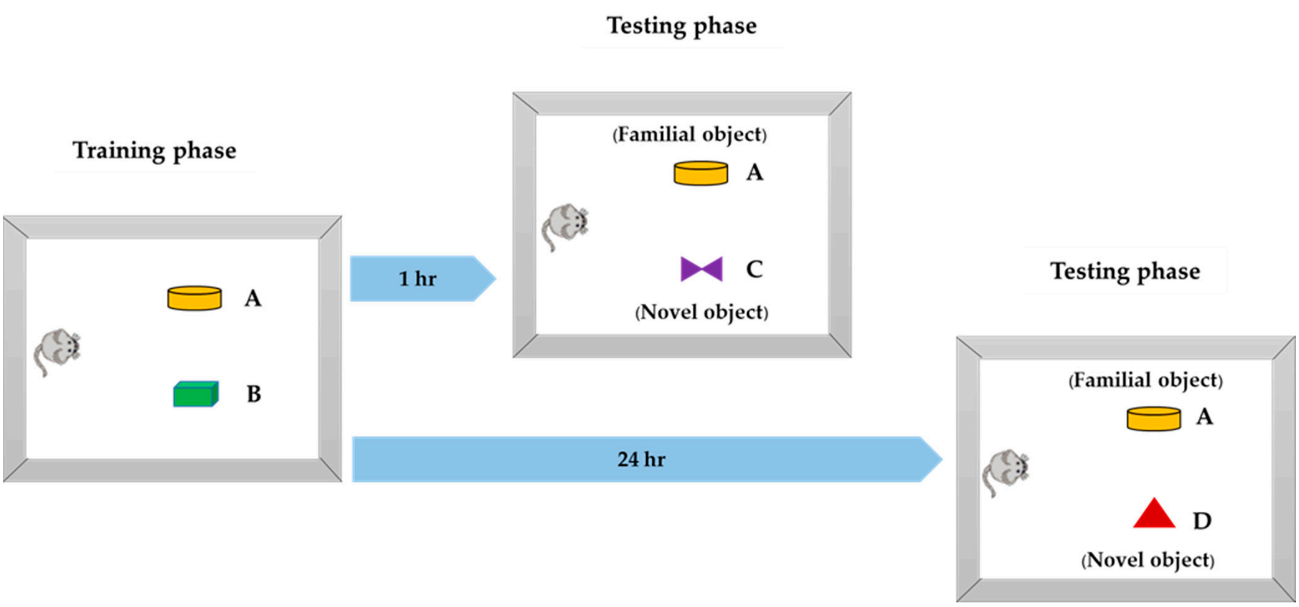

Figure S5. Schematic of object recognition test
